# Adult Mediastinal Lymphangioma Presenting As Persistent Chest Pain: A Case Report

**DOI:** 10.7759/cureus.75771

**Published:** 2024-12-15

**Authors:** Fatema M Qaher, Meaad A Husain, Maryam F Almansoor, Fatima H Abdulameer, Ahlam A Al-Ammar

**Affiliations:** 1 General Medicine, Alexandria University, Alexandria, EGY; 2 Obstetrics and Gynecology, First Health Cluster, Dammam, SAU

**Keywords:** extramedullary hematopoiesis, interventional radiology, magnetic resonance imaging, pelvic mass, splenic artery embolization

## Abstract

Mediastinal lymphangiomas are rare benign tumors arising from lymphatic system malformations, most commonly seen in pediatric populations. In adults, they are exceedingly rare and present diagnostic challenges due to nonspecific symptoms and imaging overlap with other mediastinal masses. Diagnosis is typically based on imaging, including CT and MRI, with histopathology confirming the diagnosis. Surgical resection is the treatment of choice, especially for symptomatic lesions. A 35-year-old female presented with persistent chest pain for two months. Imaging revealed a multiloculated cystic mass in the anterior mediastinum, which was consistent with a lymphangioma. The patient underwent video-assisted thoracoscopic surgery (VATS) for excision, and histopathology confirmed the diagnosis. Postoperatively, she had an uneventful recovery, and follow-up showed no recurrence at six months. This case emphasizes the importance of considering lymphangioma in the differential diagnosis of mediastinal masses, particularly in patients with persistent chest pain, and highlights the role of imaging and surgical management in achieving favorable outcomes.

## Introduction

Mediastinal lymphangiomas are rare benign tumors that arise from malformations of the lymphatic system. They are characterized by abnormal dilated lymphatic vessels and are most commonly observed in pediatric populations, accounting for up to 90% of cases diagnosed by the age of two [1,2]. In adults, mediastinal lymphangiomas are exceedingly rare and often present a diagnostic challenge due to their nonspecific clinical symptoms and radiological overlap with other mediastinal masses. These lesions are usually located in the anterior or middle mediastinum and are typically asymptomatic; however, larger lesions can cause compressive symptoms such as chest pain, dyspnea, or dysphagia [1-3].

The etiology of lymphangiomas is thought to involve congenital malformations of the lymphatic system, leading to the sequestration of lymphatic tissue that fails to communicate with normal lymphatic circulation. Secondary causes, including trauma or inflammatory conditions, are less commonly implicated [2,4]. Diagnosis often requires a combination of imaging modalities, including CT and MRI, which help differentiate lymphangiomas from other cystic mediastinal lesions such as bronchogenic cysts or thymic cysts. Histopathological examination remains the gold standard for definitive diagnosis. Surgical resection is the treatment of choice for mediastinal lymphangiomas, particularly for symptomatic or enlarging lesions. Complete excision is usually curative, with recurrence being uncommon if total resection is achieved [2-4]. This case underscores the importance of considering lymphangioma in the differential diagnosis of mediastinal masses, especially in patients presenting with persistent chest pain, and highlights the role of imaging and surgical management in achieving favorable outcomes.

## Case presentation

A 35-year-old female patient presented to the emergency department with complaints of persistent chest pain for the past two months. The pain was described as dull, non-radiating, and localized to the central chest, with no apparent aggravating or relieving factors. She denied any history of trauma, recent infections, fever, cough, hemoptysis, dyspnea, or weight loss. There was no history of smoking, alcohol consumption, or drug abuse. Her past medical history was unremarkable, with no prior surgeries, hospitalizations, or significant illnesses. Family history revealed no instances of malignancy or similar presentations.

On physical examination, the patient appeared in no acute distress. Vital signs were within normal limits: blood pressure was 120/80 mmHg, heart rate was 78 beats per minute, respiratory rate was 16 breaths per minute, and oxygen saturation was 98% on room air. Cardiovascular and respiratory examinations were unremarkable, with normal heart sounds and no murmurs or rubs. Lung auscultation revealed clear breath sounds bilaterally without adventitious sounds. Palpation of the chest elicited no tenderness. Abdominal and neurological examinations were unremarkable.

Laboratory investigations, including a complete blood count, comprehensive metabolic panel, and inflammatory markers (C-reactive protein and erythrocyte sedimentation rate), were within normal limits. D-dimer levels were checked and found to be negative, reducing the likelihood of pulmonary embolism. Troponin levels were normal, ruling out acute coronary syndrome. Initial electrocardiography showed a normal sinus rhythm without ischemic changes. A chest X-ray performed at the emergency department revealed a well-defined mediastinal mass without evidence of pleural effusion, pneumothorax, or significant lung parenchymal changes.

**Figure 1:**
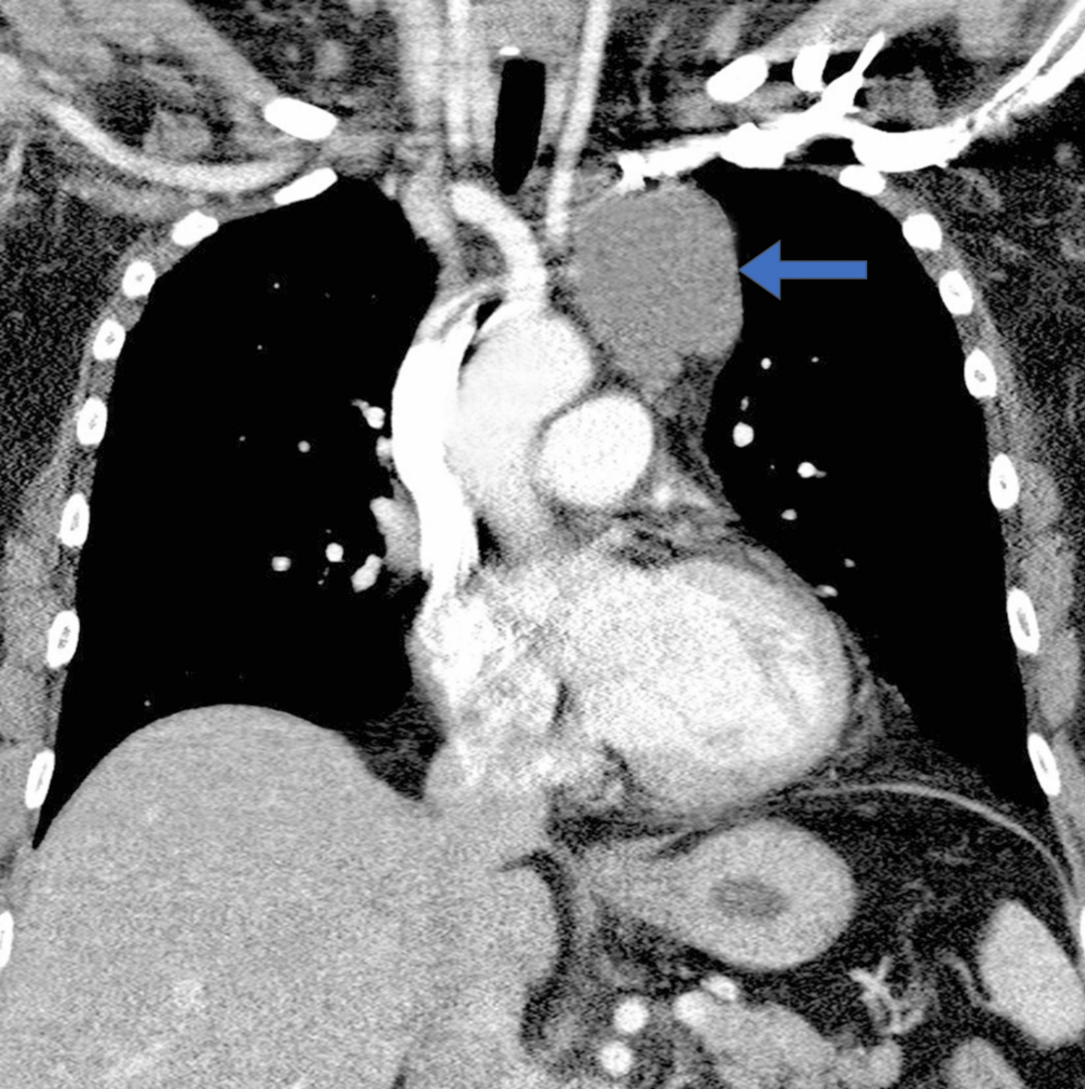
Coronal CT image demonstrates a lobulated cystic lesion (arrow) in the superior mediastinum

**Figure 2:**
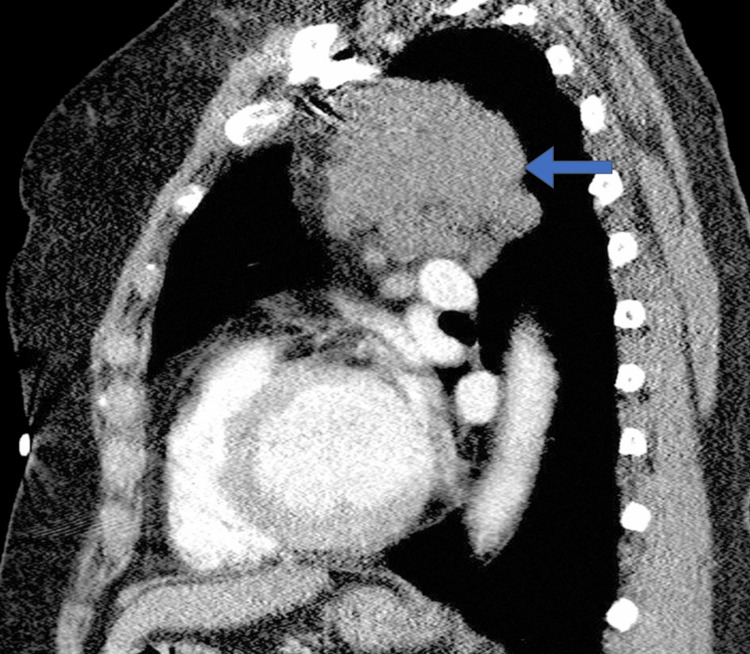
Sagittal CT image demonstrates a lobulated cystic lesion (arrow) in the superior mediastinum

Subsequent contrast-enhanced CT of the chest demonstrated a multiloculated cystic lesion measuring 6.5 cm × 4.2 cm × 5.0 cm in the anterior mediastinum. The lesion exhibited thin, enhancing septations without solid components, calcifications, or invasive features (Figures 1, 2). No significant lymphadenopathy or vascular invasion was observed. MRI was performed for further characterization, revealing hyperintense signals on T2-weighted images consistent with a cystic structure. These imaging findings were highly suggestive of a lymphangioma.

The differential diagnosis included benign mediastinal cysts (such as bronchogenic or pericardial cysts), lymphangioma, thymic cysts, and less likely, cystic teratoma or metastatic cystic lesions. Given the cystic nature, lack of solid components, and imaging characteristics, a provisional diagnosis of mediastinal lymphangioma was made.

The patient was referred to thoracic surgery for definitive management. After preoperative assessment, she underwent video-assisted thoracoscopic surgery (VATS) for excision of the lesion. Intraoperatively, the lesion was found to be encapsulated, with no adherence to adjacent structures. Complete surgical resection was achieved without complications. Histopathological examination confirmed the diagnosis of lymphangioma, showing dilated lymphatic spaces lined by endothelial cells with fibrous septa containing lymphoid aggregates. No evidence of malignancy was detected.

Postoperatively, the patient had an uneventful recovery and was discharged on the third postoperative day. At the follow-up one month later, she reported a complete resolution of chest pain and had no new symptoms. A follow-up chest X-ray showed no recurrence or residual lesion. Further imaging at six months confirmed no evidence of recurrence.

This case highlights the importance of thorough diagnostic evaluation and imaging in patients with mediastinal masses presenting as persistent chest pain. Early recognition and surgical intervention facilitated a favorable outcome, with a resolution of symptoms and no recurrence during follow-up.

## Discussion

This case of mediastinal lymphangioma presenting as persistent chest pain in an adult highlights several important considerations in the diagnosis and management of rare mediastinal masses. Mediastinal lymphangiomas, while common in pediatric populations, are exceedingly rare in adults, accounting for less than 1% of all mediastinal tumors [1-3]. Their rarity in this demographic often results in delayed diagnosis or misdiagnosis, as the clinical presentation can overlap with more common entities such as thymomas, bronchogenic cysts, or malignancies. This case emphasizes the need for a high index of suspicion and thorough diagnostic evaluation when encountering mediastinal masses in adult patients.

Advanced imaging modalities play a pivotal role in the work-up of mediastinal lymphangiomas. In this case, contrast-enhanced CT and MRI provided detailed morphological and functional insights, revealing the lesion’s cystic nature, septations, and non-invasive characteristics. The absence of metabolic activity on PET imaging further supported the benign nature of the mass [2,3]. These findings underscore the importance of a multimodal imaging approach in narrowing the differential diagnosis and guiding the management plan.

Histopathological examination remains the cornerstone for definitive diagnosis. In this case, the identification of dilated lymphatic channels lined by endothelial cells confirmed the diagnosis of lymphangioma. This histological finding, in conjunction with imaging features, is crucial for distinguishing lymphangiomas from other cystic or vascular lesions of the mediastinum [2-4].

The decision to proceed with surgical intervention in this case was based on the patient’s persistent symptoms and the risk of potential complications, such as infection, hemorrhage, or compressive effects on adjacent structures. VATS allowed for minimally invasive resection of the lesion, reducing surgical morbidity and facilitating rapid postoperative recovery. Complete surgical excision is critical to prevent recurrence, which is more likely if residual lymphatic tissue remains. The patient’s excellent clinical outcome, with a resolution of symptoms and no recurrence at the six-month follow-up, highlights the efficacy of this approach [3-6]. This case also contributes to the growing body of literature emphasizing the need for long-term follow-up in patients with resected mediastinal lymphangiomas. While recurrence is uncommon following complete resection, cases of delayed recurrence have been reported, particularly in lesions that were incompletely excised or associated with complex anatomical locations.

From a broader perspective, this case reinforces the importance of a multidisciplinary approach to the management of rare mediastinal masses. Collaboration between radiologists, thoracic surgeons, and pathologists is essential for accurate diagnosis and optimal treatment planning. Furthermore, this case serves as a reminder of the diverse presentations of mediastinal lymphangiomas and the critical role of imaging and histopathology in distinguishing them from other, potentially more aggressive, mediastinal pathologies [2,4].

## Conclusions

In conclusion, mediastinal lymphangiomas, though rare, should remain part of the differential diagnosis when evaluating mediastinal masses, particularly in symptomatic patients. Early diagnosis and definitive surgical management are key to achieving favorable outcomes. This case illustrates the successful integration of advanced imaging, meticulous surgical technique, and comprehensive follow-up in the management of a rare yet significant condition.
